# Antiviral Bioactive Compounds of Mushrooms and Their Antiviral Mechanisms: A Review

**DOI:** 10.3390/v13020350

**Published:** 2021-02-23

**Authors:** Dong Joo Seo, Changsun Choi

**Affiliations:** 1 Department of Food Science and Nutrition, College of Health and Welfare and Education, Gwangju University 277 Hyodeok-ro, Nam-gu, Gwangju 61743, Korea; sdj0118@gwangju.ac.kr; 2 Department of Food and Nutrition, School of Food Science and Technology, College of Biotechnology and Natural Resources, Chung-Ang University, 4726 Seodongdaero, Daeduck-myun, Anseong-si, Gyeonggi-do 17546, Korea

**Keywords:** mushroom, bioactive compound, virus, infection, antiviral mechanism

## Abstract

Mushrooms are used in their natural form as a food supplement and food additive. In addition, several bioactive compounds beneficial for human health have been derived from mushrooms. Among them, polysaccharides, carbohydrate-binding protein, peptides, proteins, enzymes, polyphenols, triterpenes, triterpenoids, and several other compounds exert antiviral activity against DNA and RNA viruses. Their antiviral targets were mostly virus entry, viral genome replication, viral proteins, and cellular proteins and influenced immune modulation, which was evaluated through pre-, simultaneous-, co-, and post-treatment in vitro and in vivo studies. In particular, they treated and relieved the viral diseases caused by herpes simplex virus, influenza virus, and human immunodeficiency virus (HIV). Some mushroom compounds that act against HIV, influenza A virus, and hepatitis C virus showed antiviral effects comparable to those of antiviral drugs. Therefore, bioactive compounds from mushrooms could be candidates for treating viral infections.

## 1. Mushrooms and Their Compounds

There are approximately 12,000 known species of mushrooms worldwide, of which at least 2000 species are edible [1]. *Lentinus* (*Lentinula*), *Auricularia*, *Hericium*, *Grifola*, *Flammulina*, *Pleurotus*, *Lactarius*, *Pisolithus*, *Tremella*, *Russula*, *Agaricus*, and *Cordyceps* are well-known edible mushroom species [2]. They are consumed in their natural form or as food supplements. Mushrooms contain moisture (85–95%), carbohydrates (35–70%), protein (15–34.7%), fat (<10%), minerals (6–10.9%), nucleic acids (3–8%), and very low levels of vitamins [1,2]. Since mushrooms have carbohydrates, fiber, protein, essential amino acids, unsaturated fatty acids, minerals, vitamins, and low calories, they are recognized as a healthy food with nutritional benefits [2].

Several bioactive metabolites present in mushrooms are polysaccharides, terpenoids, and phenolic compounds, including flavonoids, glycoproteins, polyketides, steroids, and alkaloids [2]. They have beneficial effects for human health [1,2]. Among them, polysaccharides and terpenoids are important bioactive metabolites. Polysaccharides, such as glucan, β-glucan, mannoglucan, heteroglycan, galactomannan, and lentinan, exhibit immunomodulatory, antioxidant, anti-inflammatory, antitumor, and antiviral activities. Terpenoids affect adipocyte differentiation and exhibit antimicrobial, anticholinesterase, antiviral, anti-inflammatory, and nitric oxide (NO) production inhibitory activity. Phenolic compounds showed NO production inhibition, antiviral, anti-inflammatory, and antioxidant activities [1,2].

## 2. Replication Steps of Viruses and Antiviral Targets

The viral replication cycle involves attachment, penetration, uncoating, replication, assembly, and release steps [3]. Viruses attach to cells through the interaction of viral capsid or glycoprotein with the cell receptor and enter host cells. Then, viral nucleic acid is released, which is used for replication. Viral replication is dependent on the viral genome. Most DNA viral genomes are replicated and transcribed using cell enzymes, such as DNA-dependent DNA polymerase and RNA polymerase II, into the nucleus. However, DNA viruses with large genomes (e.g., vaccinia virus) encode their own enzymes responsible for replication in the cytoplasm. RNA viruses usually use the viral genome as a template for mRNA synthesis. They replicate and transcribe genomes using viral RNA-dependent RNA polymerase (RdRp). Some RNA and DNA viruses involve reverse transcription in the viral replication cycle. HIV and HBV are representative reverse transcribing viruses that synthesize DNA from RNA using RNA-dependent DNA polymerase. Viral mRNA is then translated into proteins in the cell cytoplasm [3]. Viral proteins with a replicated viral genome are packaged to new virions. Viruses are released through cell lysis, budding, and exocytosis.

There are therapeutic agents or vaccines against human viruses such as herpes simplex virus (HSV), influenza virus, human immunodeficiency virus (HIV), hepatitis C virus (HCV), and enteroviruses; however, they may have adverse effects, including drug resistance [3,4,5,6,7,8]. Patients infected with HIV showed resistance to nucleoside or non-nucleoside reverse transcriptase inhibitors [4,5]. Ledipasvir acetonate and sofosbuvir for HCV infection led to interstitial lung disease [6]. Therefore, promising antiviral compounds from natural sources need to be developed as safe agents.

Mushroom compounds reduce viral infection by mostly targeting viral entry, genome replication, viral enzymes, viral proteins, and cellular proteins, and influence immune modulation (Tables 1–3). The antiviral activity of therapeutic agents was evaluated by the time-of-addition assay as follows: pre-, simultaneous-, co-, and post-treatment [9,10,11,12,13,14,15,16,17,18,19,20,21,22,23,24,25,26,27,28,29,30,31,32,33,34,35,36,37,38,39,40,41]. Prevention of viral infection was identified by pretreatment with antiviral agents in cells. The antiviral effect of simultaneous treatment means that the initial stages of replication steps were inhibited by treatment with both viruses and antiviral agents into cells simultaneously. The cotreatment effect was evaluated to observe the virucidal effect and the interaction of cell receptors with viruses treated with antiviral agents. The inhibition of viral replication was evaluated by treatment with antiviral agents after viral infection in cells.

## 3. Antiviral Activity of Mushroom Compounds against Viruses

### 3.1. Enveloped DNA Viruses

#### Herpes Simplex Viruses

Herpes simplex virus (HSV) is an enveloped double-stranded DNA virus belonging to the Herpesviridae family. HSV is characterized into two serotypes: HSV-1 and HSV-2. HSV-1 can cause gingivostomatitis, herpes labialis, encephalitis, herpes keratitis, and genital herpes, whereas HSV-2 is associated with genital infection, neonatal herpes, and herpetic paronychia [42,43]. The viral genome encodes >80 different proteins and divides into immediate early (IE or α), early (E or β), and late (L or γ) genes. IE genes are immediately translated to IE proteins. IE proteins, including ICP0, ICP4, and ICP27, are transcriptional regulators essential for the transcription of E and L genes. E genes encode the proteins responsible for nucleotide and DNA metabolism (UL2, UL12, UL23, UL40, and UL50), viral replication (UL5, UL30, and UL42), and protein modification (US3). L genes, such as UL10, UL18, UL27, and US4, are translated into viral structure proteins, such as glycoprotein and capsid protein [7,42].

The current antiviral agents against HSV are nucleoside analogs, including acyclovir, famciclovir, valacyclovir, penciclovir, and trifluridine [43]. Acyclovir is the most popular anti-HSV drug used for genital and labial herpes. It is phosphorylated by viral thymidine kinase and cellular kinases and is incorporated into viral DNA, which inhibits viral replication by HSV DNA polymerase. Other antiviral agents are trifluridine for herpes keratitis and docosanol for recurrent labial herpes; however, infection of HSV resistant to nucleoside analogs is possible in immunocompromised patients. Intravenous foscarnet, a pyrophosphate analogue, is used as a second-line therapy for HSV infection resistant to nucleoside analogs. However, it induces nephrotoxicity and electrolyte disorders as an adverse reaction [43]. Other agents or vaccines should be developed to treat HSV. *Boletus edulis*, *P. ostreatus*, *L. edodes*, *Phellinus pini*, *Ganoderma pfeifferi*, *Rozites caperata*, *A. brasiliensis*, and *G. lucidum* have shown an antiherpetic effect (Table 1) [9,10,11,12,13,14,15,16,17].

**Figure 1:**
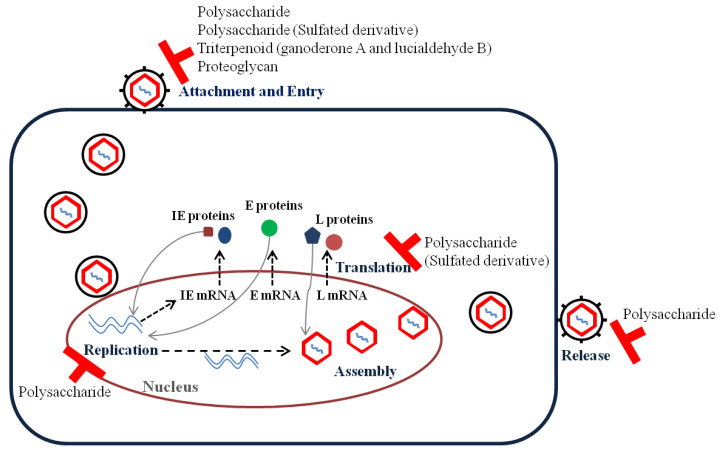
Inhibitory stages of bioactive compounds against herpes simplex virus.

Mushroom compounds, including polysaccharide, sulfated polysaccharide, proteoglycan, peptide RC28, triterpenoid (ganoderone A, lucialdehyde B, and ganodermadiol), have shown pre-, simultaneous-, co-, and post-treatment effects according to the type of mushroom (Table 1). They may be effective on all viral replication steps, including entry, uncoating, replication, assembly, and release (Figure 1).

Sulfated polysaccharide from *A. brasiliensis* inhibited viral attachment, penetration, and cell-to-cell spread into African green monkey kidney (Vero) cells [14]. Indeed, it decreased the expression of ICP27, UL42, gB, and gD proteins, which suggests that sulfated polysaccharide affected viral glycoprotein, replication, and transcription of E and L genes. An in vivo study showed that intravaginal pretreatment with sulfated polysaccharide reduced genital infection by HSV-2 in mice. In addition, sulfated polysaccharide was effective on cutaneous disease in mice when it was orally administered after HSV-1 infection [15]. Its post-treatment did not relieve keratitis by HSV-1, while peptide RC28 from *R. caperata* delayed keratitis [13]. Interestingly, the antiherpetic activity of peptide RC28 was as effective as that of ganciclovir in mice. As sulfated polysaccharide and peptide RC28 from mushrooms exerted antiviral effect both in vitro and in vivo against HSV, these have potential as therapeutic agents.

### 3.2. Enveloped RNA Viruses

#### 3.2.1. Influenza Viruses

Influenza virus, an enveloped virus that belongs to the *Orthomyxoviridae* family, is classified into types A, B, C, and D [44]; it is divided into various serotypes based on glycoproteins. The genome of influenza A and B viruses comprises eight single-stranded negative-sense RNA segments, while that of influenza C and D viruses contains seven segments. The viral genome is about 13.5 kb and encodes hemagglutinin, neuraminidase (NA), nucleocapsid protein, RdRp, matrix proteins (M1 and M2), and nonstructural proteins (NS1 and NS2). Influenza A and B viruses are associated with seasonal flu epidemics. To date, oseltamivir (Tamiflu), zanamivir (Relenza), peramivir (Rapivab), and laninamivir (Inavir) have been used as NA inhibitors. Baloxavir marboxil, a novel cap-dependent endonuclease inhibitor, is an influenza therapeutic agent approved in 2018. However, there have been issues reported regarding its adverse effects, toxicity, and the emergence of resistant viruses [45,46].

Compounds from *P. pulmonarius*, *C. militaris*, *L. edodes*, *P. baumii*, *P. ignarius*, *G. pfeifferi*, and *P. linteus* exert antiviral effects against influenza viruses: polysaccharide fraction from *P. pulmonarius*; acidic polysaccharide from *C. militaris*; peptidomannan from *L. edodes*; polyphenols (hispidin, inoscavin A, davallialactone, and phelligridin D) from *P. baumii*; polyphenols, pyrone (3-hydroxy-2-methyl-4-pyrone), and sesquiterpenoid from *P. ignarius*; triterpenoid (ganodermadiol and lucidadiol) and triterpene (applanoxidic acid G) from *G. pfeifferi*; and other organic compounds, including inotilone and 4-(3,4-dihydroxyphenyl)-3-buten-2-one, from *P. linteus* (Table 2) [12,18,19,20,21,22,23].

Polyphenols (hispidin, hypholomine B, inoscavin A, davallialactone, and phelligridin D), sesquiterpenoid, inotilone, and 4-(3,4-dihydroxyphenyl)-3-buten-2-one targeted viral NA [20,22,23]. It has been identified that the hydroxyl group of sesquiterpenoid interacts with the amino acid (Asn 170) of NA. In addition, sesquiterpenoid and zanamivir are bound to different sites of NA. Polyphenols, sesquiterpenoid, pyrone, ganodermadiol, lucidadiol, and applanoxidic acid G inhibited the cytopathic effect of influenza A virus [12,22,23]; interestingly, their 50% inhibitory concentration (IC_50_) was lower than that of the antiviral drug zanamivir. Inotilone and 4-(3,4-dihydroxyphenyl)-3-buten-2-one were more potent than oseltamivir (Tamiflu) against influenza A virus [20].

Polysaccharide fractions of water and ethanol extracts of *P. pulmonarius* exerted antiviral activity against influenza A virus (H1N1pdm) [18]. As polysaccharide fractions only showed viral reduction by post-treatment into cells, it may block viral replication, assembly, and release. There was a report that acidic polysaccharide from *C. militaris* affected immune enhancement [19]. Acidic polysaccharide decreased the influenza A virus (H1N1) titer in the bronchoalveolar region and lung of mice, and increased tumor necrosis factor (TNF)-α and interferon (IFN)-γ levels in the blood, bronchoalveolar lavage fluid (BALF), and lung of mice. Additionally, it enhanced NO, inducible nitric oxide synthase, interleukin (IL)-10, and proinflammatory cytokines such as IL-1β, IL-6, and TNF-α in RAW 264.7 cells [19]. Oral administration and the intraperitoneal injection of peptidomannan increased the survival rate and elaborated the IFN level in the serum of influenza A virus (H2N2)-infected mice [21]. In addition, it reduced lung consolidation and viral titer in the lung tissue of mice. However, there was no inhibition of the virus by peptidomannan in vitro. Acidic polysaccharide and peptidomannan seemed to reduce viral infection through immune enhancement.

#### 3.2.2. Human Immunodeficiency Virus

Human immunodeficiency virus (HIV) belonging to the *Retroviridae* family is an enveloped virus containing positive-sense, single-stranded RNA [3]. HIV genes encode 15 viral proteins, including viral structural proteins, such as viral enzymes and envelope, essential regulatory elements, and accessory regulatory proteins. There are two types of HIV: HIV-1 and HIV-2. HIV-1 is more closely associated with the worldwide immunodeficiency syndrome epidemic than HIV-2. As HIV infects CD4 T cells, macrophages, and dendritic cells responsible for immune responses, it compromises the defense against opportunistic infections and tumors [3].

There are therapeutic agents that target viral reverse transcriptase, protease, viral attachment and fusion, and integrase [3]. Nucleoside analogs, such as azidothymidine (AZT) and lamivudine, bind to reverse transcriptase and inhibit viral reverse transcription. Nevirapine is a benzodiazepine non-nucleoside inhibitor targeting reverse transcriptase. Ritonavir on the active site of protease blocks the maturation of HIV into the infectious virion. Maraviroc inhibits viral attachment to the CCR5 receptor on cell surface. Enfuvirtide inhibits viral fusion on host cells by interacting with gp41 of the HIV envelope glycoprotein. However, they can induce the gene mutation of reverse transcriptase, protease, and the envelope of drug-resistant HIV and thus cause adverse effects [3].

Anti-HIV mushroom compounds have been derived from *P. abalonus*, *Coriolus versicolor*, *A. bisporus*, *P. citrinopileatus*, *L. edodes*, *P. ostreatus*, *R. paludosa*, and *Tricholoma giganteum* (Table 2) [24,25,26,27,28,29,30,31,32,33]. Polysaccharide, polysaccharopeptide, lectin, lentin, ubiquitin-like protein, peptide, and laccase were identified to mainly target HIV reverse transcriptase [25,26,27,28,29,30,31,32]. Interestingly, polysaccharide, lectin, lentin, and laccase from *P. abalonus*, *P. citrinopileatus*, *L. edodes*, and *T. giganteum* showed IC_50_ of 0.1–2.2 μM against HIV-1 [25,28,29,32]. Anti-HIV drugs, such as AZT, dideoxycytidine and dideoxyinosine, showed IC_50_ of 0.03–0.5 μM, 0.5–1.5 μM, and 2–10 μM, respectively, against HIV-1 [3]. Therefore, compounds derived from mushrooms could be antiviral agents comparable to antiviral drugs.

Polysaccharopeptide from *C. versicolor* inhibited HIV reverse transcriptase and glycohydrolase (β-glucuronidase) involved in the glycosylation of the HIV envelope to neutralize antibody epitopes [26]. It also interfered with the interaction of HIV-l gp120 with the CD4 receptor. Polysaccharopeptide modified with chlorosulfonic acid inhibited both glycohydrolase (α-glucosidase) and HIV reverse transcriptase [27]. Polysaccharopeptide can interfere with HIV entry and replication by inhibiting reverse transcription.

Immune Assist 24/7™ (*L. edodes*, *G. frondosa*, *G. lucidum*, *Trametes versicolor*, *C. sinensis*, and *A. brasiliensis*) is commercially available and comprises multiple polysaccharides and heteropolysaccharides [24]. It increased CD4+ T-lymphocytes at 30 and 60 days when HIV-infected patients were administered 2.4 g/day of Immune Assist 24/7™ for 60 days. Nebrodeolysin from *P. nebrodensis* reduced syncytia formation in HIV-infected human T-lymphocytes with a 50% effective concentration (EC_50_) of 2.4 pM [33]. Polysaccharide, heteropolysaccharide, and nebrodeolysin may influence viral replication in T-lymphocytes.

#### 3.2.3. Hepatitis C Virus

Hepatitis C virus (HCV), which is an enveloped, positive-stranded RNA virus, is a member of the *Flaviviridae* family that causes chronic hepatitis and hepatocellular carcinoma [47]. Although there is no available vaccine to date, antiviral drugs are used to fight HCV infection [3,8]. Ribavirin is a guanosine analog used to treat RNA virus infection. Its antiviral activity is conferred by its inhibition of the inosine monophosphate dehydrogenase used for the production of viral RNA. Its combination with IFN-α showed synergistic antiviral effects [3]. There are direct acting antivirals (DAAs) to treat HCV infection [8]. DAAs target NS3/4A protease, NS5A, and RdRp that are nonstructural proteins of HCV. NS3/4A protease inhibitors (boceprevir, telaprevir, simeprevir, paritaprevir, asunaprevir, and grazoprevir) bind to NS3 catalytic site and inhibit the cleavage of viral polyprotein. NS5A inhibitors (daclatasvir, ledipasvir, ombitasvir, elbasvir, velpatasvir, and pibrentasvir) block viral replication by binding to domain I of the NS5A protein. NS5B RdRp inhibitors, such as sofosbuvir and dasabuvir, block viral transcription and gene replication. However, these antiviral agents result in adverse effects and drug resistance [3,8].

Laccase and tyrosinase enzymes from *P. ostreatus* and *A. bisporus*, respectively, have antiviral effects against HCV (Table 2) [34,35]. Laccase (2.0 and 2.5 mg/mL) from *P. ostreatus* inhibited HCV when peripheral blood cells (PBMCs) and hepatoma HepG2 cells were pretreated with it [34]. The inhibition of HCV cotreated with 2.0 and 2.5 mg/mL of laccase was identified through the immunostaining assay of PBMCs and HepG2 cells. Additionally, 0.75 and 1.5 mg/mL of laccase showed a post-treatment effect on HCV-infected HepG2 cells. This means that laccase from *P. ostreatus* can block viral entry and replication on PBMCs and HepG2 cells [34]. Tyrosinase (molecular weight, 50 kDa) from *A. bisporus* inhibited viral replication into replicon-containing Huh-5-2 cells [35]. Interestingly, the EC50 of tyrosinase was 1.2–2.5 μg/mL, while that of ribavirin was 10 μg/mL. In addition, tyrosinase targeted viral nonstructural proteins, such as NS3, NS4A, and NS5A, which are essential for viral replication. It interacted with the amino terminal of proteins and altered the catalytic activity of NS3 [35].

### 3.3. Non-Enveloped RNA Viruses

#### 3.3.1. Norovirus

Human norovirus (HuNoV) is a non-enveloped, positive-sense, single-stranded RNA virus that belongs to the family *Caliciviridae* [48]. HuNoV is transmitted via the fecal–oral route and causes gastroenteritis. It is resistant in water, food items, the environment (e.g., acidic, dry, and chilled conditions), and on various surfaces. As no antiviral drugs or vaccines are available for this in humans, several studies have reported antiviral food extracts and compounds [49]. *Inonotus obliquus* exhibited antiviral activity against the norovirus surrogates murine norovirus (MNV) and feline calicivirus (FCV) (Table 3) [36,37].

**Figure 2:**
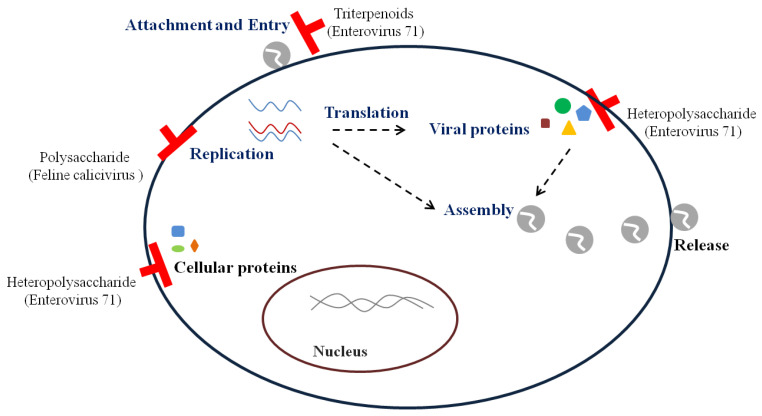
Inhibitory mechanisms of antiviral bioactive compounds against non-enveloped viruses.

*I. obliquus* significantly decreased MNV-1 titers by 91.67 ± 3.36% when pretreated in RAW 264.7 cells [36]. A polysaccharide from *I. obliquus* reduced FCV-F9 and field strains of FCV [37]. It inhibited CPE and viral RNA level through pre-, co-, post-, and simultaneous treatment against FCV-F9 in Crandell feline kidney (CRFK) cells. Moreover, the inhibition of FCV expressing green fluorescent protein incorporated into VP1 was observed in CRFK cells pretreated with the polysaccharide, which suggests that the polysaccharide influences viral replication (Figure 2). Since *I. obliquus* and its polysaccharide reduced norovirus surrogates by pre-, co-, post-, and simultaneous treatment into cells, they are likely to inhibit several replication steps.

#### 3.3.2. Enterovirus 71

Enterovirus, which is a member of the *Picornaviridae* family, is a non-enveloped RNA virus with positive-sense genome [50]. The genus Enterovirus includes 15 species: enterovirus A–L and rhinovirus A–C. Enterovirus species include serotypes, such as enterovirus (EV), rhinovirus, coxsackievirus (CV), echovirus, poliovirus (PV), simian virus, and baboon enterovirus. These are transmitted and infected from one person to another, via the fecal–oral route, and via air [50,51]. Viral infection causes a wide range of diseases, including asymptomatic infection, common cold, enteroviral vesicular stomatitis, encephalitis, meningitis, paralysis, and myocarditis.

Among enteroviruses, PV was eradicated with oral polio vaccines [51]. Antiviral candidates against PV and EV 71 were recently developed. Antiviral targets were viral capsid, proteases, 3A and 2C proteins, RNA polymerase, and cell proteins associated with replication and assembly. Pleconaril, pirodavir, pirodavir analogs, and pyridyl imidazolidinones bind to a hydrophobic pocket of the viral capsid and inhibit its attachment to cells. Peptidomimetic rupintrivir and rupintrivir analogs are inhibitors of proteases that cleave viral polyprotein. Enviroxime, benzimidazoles, and N(6)-benzyladenosine target viral proteins 3A and 2C and inhibit intracellular transport and encapsidation of virions. Ribavirin is widely used for RNA viruses and exerts antiviral activity by targeting RdRp. Brefeldin A and geldenamycin inhibit cell proteins involved in viral replication and assembly. However, as their antiviral activity has only been observed in vitro, clinical trials or in vivo studies are required to use them [51].

Heteropolysaccharide and triterpenoids reportedly exhibit antiviral action against EV 71 (Table 3) (Figure 2) [38,39]. Heteropolysaccharide from *G. frondosa* exerted pre-, simultaneous-, and post treatment effects in Vero cells [38]. The pretreatment with the heteropolysaccharide suppressed the expression of the capsid protein VP1. In addition, it decreased caspase-3 activation and IκBα, which are known to inhibit nuclear factor kappa B (NF-κB) transcription; this may reduce cell death by apoptosis induced by EV71 and influence the expression of proinflammatory cytokines by NF-κB. Heteropolysaccharide from *G. frondosa* could block the expression of capsid protein and reduce viral infections by suppressing apoptosis.

Triterpenoids from *G. lucidum* reduced the viral RNA level by pre- and cotreatment in human rhabdomyosarcoma (RD) cells [39]. The interaction of viral capsid with triterpenoids was observed through a molecular docking program that calculated the energy change at the binding site. The possible antiviral mechanism of triterpenoids was the inhibition of viral penetration into cells by blocking viral entry.

#### 3.3.3. Poliovirus and Coxsackievirus

Polysaccharide derived from *A. brasiliensis* and *L. edodes* exerted antiviral activity against PV-1 (Table 3) [40,41]. Simultaneous treatment of polysaccharide and PV-1 reduced the viral titer into human larynx epithelial cells carcinoma (HEp-2) cells, whereas pre-, co-, and post-treatment effects were not observed. Polysaccharide from *P. pini* showed the reduction of the coxsackievirus B3 (CVB3) titer when it was simultaneously treated with CVB3 in HeLa cells (Table 3) [10]. Therefore, polysaccharides from *A. brasiliensis*, *L. edodes*, and *P. pini* may influence the initial step of viral replication.

## 4. Conclusions

As mushrooms have bioactive compounds and high nutritional value, they provide a broad range of health benefits. Bioactive compounds reportedly showing antiviral properties are polysaccharides, carbohydrate-binding proteins (polysaccharopeptide and peptidomannan), proteins (ubiquitin-like protein, nebrodeolysin, lectin, and lentin), peptides, enzymes (laccase and tyrosinase), polyphenols, triterpenes, triterpenoids, and several other compounds. They reportedly inhibit viral entry, replication, viral enzyme, the expression of viral proteins, and cellular proteins, and enhance immunity against HSV-1, HSV-2, influenza A virus, HIV, HCV, FCV, and EV71. In addition, polysaccharide, sulfated polysaccharide, acidic polysaccharide, peptidomannan, and peptide relieved viral diseases caused by HSV, influenza virus, and HIV. Antiviral mechanisms of mushroom compounds were well defined against enveloped viruses; however, that still needs to be evaluated against non-enveloped viruses such as NoV and enteroviruses. Bioactive metabolites derived from mushrooms could be considered as potential antiviral candidates against DNA and RNA viruses.

## Figures and Tables

**Table 1:** Anti-herpes simplex virus compounds of mushrooms and their antiviral targets.

Viruses		Mushrooms	Compounds	Targets or Effects	References
Enveloped DNA Viruses					
Herpes simplex virus type 1 (HSV-1)	dsDNA	*Boletus edulis*, *Pleurotus ostreatus*, and *Lentinus edodes*	Polysaccharide fraction	Pre- and post-treatment effect	[9]
HSV-1		*Phellinus pini*	Polysaccharide	Simultaneous-treatment effect	[10]
HSV-1		*Ganoderma pfeifferi*	Triterpenoid (ganoderone A and lucialdehyde B)	Pre-treatment effect	[11]
HSV-1		*G. pfeifferi*	Triterpenoid (ganodermadiol)	Protection of cells	[12]
HSV-1		*Rozites caperata*	Peptide RC28	Keratitis (in vivo)	[13]
HSV-1 and HSV-2		*Agaricus brasiliensis*	Polysaccharide (Sulfated derivative)	Viral attachment and penetration, cell-to-cell spread, and the expression of ICP27, UL42, gB, and gD proteins	[14]
HSV-1 and HSV-2		*A. brasiliensis*	Polysaccharide (Sulfated derivative)	Cutaneous and genital infection (in vivo)	[15]
HSV-1 and HSV-2		*G. lucidum*	Proteoglycan	Pre- and co-treatment effect	[16,17]

**Table 2:** Mushroom compounds and their antiviral targets against enveloped RNA viruses.

Viruses		Mushrooms	Compounds	Targets or Effects	References
Enveloped RNA viruses					
Influenza A virus (H1N1pdm09)		*Pleurotus pulmonarius*	Polysaccharide fraction	Post-treatment effect	[18]
Influenza A virus (H1N1)	Segmented (-)ssRNA	*Cordyceps militaris*	Acidic Polysaccharide	Decreased viral titer in bronchoalveolar and lung, increased TNF-α and IFN-γ in mice, and enhanced NO, iNOS, IL-1β, IL-6, IL-10, and TNF-α in cells	[19]
Influenza A virus (H1N1 and WS/33)		*Phellinus linteus*	Inotilone and 4-(3,4-dihydroxyphenyl)-3-buten-2-one	Neuraminidase (NA) Simultaneous-treatment effect	[20]
Influenza A virus (H2N2)		*Lentinus edodes*	Peptidomannan	Decreased viral titer and lung consolidation in lung tissue, elaborated IFN level in serum, and increased survival in mice	[21]
Influenza A virus (H1N1, H5N1, and H3N2)		*P. baumii*	Polyphenols (hispidin, hypholomine B, inoscavin A, davallialactone, and phelligridin D)	NA	[22]
Influenza A virus (H5N1)		*P. ignarius*	Sesquiterpenoid	NA	[23]
			Sesquiterpenoid Pyrone Polyphenols	Post-treatment effect	
Influenza A virus		*Ganoderma pfeifferi*	Triterpenoid (ganodermadiol and lucidadiol) Triterpene (applanoxidic acid G)	Protection of cells	[12]
Human immunodeficiency virus (HIV)	(+)ssRNA	Immune Assist 24/7™(*L. edodes*, *G. frondosa*, *G. lucidum*, *Trametes versicolor*, *C. sinensis*, and *A. brasiliensis*)	Multiple polysaccharide and heteropolysaccharide	Increased CD4+ T-lymphocyte	[24]
HIV-1		*P. abalonus*	Polysaccharide	Reverse transcriptase	[25]
HIV-1		*Coriolus versicolor*	Polysaccharopeptide	Interaction of HIV-l gp120 with CD4, reverse transcriptase, and glycohydrolase	[26]
HIV-1		*C. versicolor*	Polysaccharopeptide (modified with chlorosulfonic acid)	Reverse transcriptase and glycohydrolase	[27]
HIV-1		*Agaricus bisporus*	Lectin	Reverse transcriptase	
HIV-1		*P. citrinopileatus*	Lectin	Reverse transcriptase	[28]
HIV-1		*L. edodes*	Lentin	Reverse transcriptase	[29]
HIV		*P. ostreatus*	Ubiquitin-like Protein	Reverse transcriptase	[30]
HIV-1		*Russula paludosa*	Peptide	Reverse transcriptase	[31]
HIV-1		*Tricholoma giganteum*	Laccase	Reverse transcriptase	[32]
HIV-1		*P. nebrodensis*	Nebrodeolysin	HIV-induced syncytia formation in cells	[33]
Hepatitis C virus (HCV)	(+)ssRNA	*P. ostreatus*	Laccase	Pre-, co-, post- treatment effect	[34]
HCV		*A. bisporus*	Tyrosinases	NS3, NS4A, and NS5A	[35]

**Table 3:** Mushroom compounds and their antiviral targets against non-enveloped RNA viruses.

Viruses		Mushrooms	Compounds	Targets or Effects	References
Non-enveloped RNA Viruses					
Murine norovirus Feline calicivirus (FCV)	(+)ssRNA	*Inonotus obliquus*	-	Pre-treatment effect	[36]
FCV		*I. obliquus*	Polysaccharide	Pre-, co-, post-, and simultaneous-treatment effect Viral replication	[37]
Enterovirus 71 (EV71)	(+)ssRNA	*Grifola frondosa*	Heteropolysaccharide	Pre-, simultaneous-, and post- treatment effect VP1, caspase-3, and IκBα	[38]
EV71		*Ganoderma lucidum*	Triterpenoids	Pre- and co- treatment effect Adsorption	[39]
Poliovirus type 1 (PV-1)	(+)ssRNA	*Agaricus brasiliensis*	Polysaccharide	Simultaneous-treatment effect	[40]
PV-1		*Lentinula edodes*	Polysaccharide	Simultaneous-treatment effect	[41]
Coxsackie virus B3	(+)ssRNA	*Phellinus pini*	Polysaccharide	Simultaneous-treatment effect	[10]
